# Dissection of Chemical Composition and Associated Gene Expression in the Pigment-Deficient Tea Cultivar ‘Xiaoxueya’ Reveals an Albino Phenotype and Metabolite Formation

**DOI:** 10.3389/fpls.2019.01543

**Published:** 2019-11-27

**Authors:** Na-Na Li, Jian-Liang Lu, Qing-Sheng Li, Xin-Qiang Zheng, Xin-Chao Wang, Lu Wang, Yu-Chun Wang, Chang-Qing Ding, Yue-Rong Liang, Ya-Jun Yang

**Affiliations:** ^1^Key Laboratory of Tea Biology and Resources Utilization, Ministry of Agriculture and Rural Affairs, National Center for Tea Plant Improvement, Tea Research Institute, Chinese Academy of Agricultural Sciences, Hangzhou, China; ^2^Tea Research Institute, Zhejiang University, Hangzhou, China

**Keywords:** *Camellia sinensis*, albino, chloroplast, caffeine, amino acid, catechins

## Abstract

The tea cultivar ‘Xiaoxueya’, a temperature-sensitive albino mutant, is a rare tea germplasm because of its highly enriched amino acid content and brisk flavour. In comparison with green leaf tissues of ‘Xiaoxueya’, albino leaves show significant deficiency in chlorophylls and carotenoids and severely disrupted chloroplasts. Furthermore, the accumulation of quality-related secondary metabolites is altered in ‘Xiaoxueya’ albino leaf, with significantly increased contents of total amino acids, theanine, and glutamic acid and significantly decreased contents of alkaloids, catechins, and polyphenols. To uncover the molecular mechanisms underlying albinism and quality-related constituent variation in ‘Xiaoxueya’ leaves, expression profiles of pivotal genes involved in the biosynthetic pathways of pigments, caffeine, theanine, and catechins were investigated by quantitative real-time PCR technology. The results revealed that suppressed expression of the chloroplast-localized 1-deoxy-D-xylulose-5-phosphate synthase genes *DXS1* and *DXS2* involved in the 2-C-methyl-D-erythritol-4-phosphate (MEP) pathway and protochlorophyllide oxidoreductase genes *POR1* and *POR2* involved in the chlorophyll biosynthetic pathway is responsible for the pigment deficiency in ‘Xiaoxueya’ albino leaf. Additionally, the low expression of the tea caffeine synthase gene (*TCS)* involved in caffeine biosynthesis and the chalcone synthase genes *CHS1*, *CHS2*, and *CHS3*, the chalcone isomerase gene *CHI*, the flavonoid 3’,5’-hydroxylase genes *F3’5’H1* and *F3’5’H2*, and the anthocyanidin reductase genes *ANR1* and *ANR2* involved in the flavonoid pathway is related to the reduction in alkaloid and catechin levels in ‘Xiaoxueya’ albino leaves.

## Introduction

The tea plant (*Camellia sinensis*) is a perennial evergreen woody plant, and its shoots are utilized as raw materials for manufacturing various types of tea products, such as green tea, black tea, and oolong tea. These tea products, especially green tea, have attracted great attention worldwide for their *umami* taste, fresh aroma, and health benefits (Butt and Sultan, 2009; Steinmann et al., 2013). As the fresh leaves from various tea cultivars differ in chemical composition (Du et al., 2006; Feng et al., 2014), the tea cultivar is an important factor influencing the sensory attributes of tea including colour, aroma, and taste of unfermented green tea. In recent years, researchers have bred chlorophyll-deficient albino tea cultivars with high levels of amino acids and moderate levels of polyphenols and caffeine to produce high-quality green tea products (Ma et al., 2012; Li et al., 2016c). There are two types of chlorophyll-deficient tea cultivars grown in China: temperature-sensitive albino tea cultivars with white leaves, such as ‘White leaf No.1’ and ‘Xiaoxueya’ (Du et al., 2008; Li et al., 2011), and light-sensitive albino tea cultivars with yellow leaves, such as ‘Huangjinya’, ‘Zhonghuang 2’, and ‘Yujinxiang’ (Wang et al., 2014a; Li et al., 2016c; Liu et al., 2017).

‘White leaf No.1’ was the first temperature-sensitive albino tea cultivar used in China, and it has been widely grown in Zhejiang, Jiangsu, and Guizhou provinces. Many studies have attempted to reveal the physiological and biochemical characteristics of ‘White leaf No.1’. For example, the contents of amino acids, including theanine, were found to be significantly higher in young albino shoots of ‘White leaf No.1’ than in normal tea cultivars with green leaves (Du et al., 2006; Li et al., 2016b). Conversely, the levels of pigment components, including chlorophyll *a*, chlorophyll *b*, and carotenoids, as well as quality-related metabolites, including catechins and caffeine, were significantly decreased in the chlorophyll-deficient albino leaves of ‘White leaf No.1’ (Du et al., 2006; Wei et al., 2012; Feng et al., 2014; Li et al., 2015). Furthermore, ultrastructural studies have revealed that the albino leaves of ‘White leaf No.1’ contain aberrant chloroplasts lacking grana and thylakoids (Li et al., 2011; Zhu et al., 2016). Li et al. (2011) used two-dimensional electrophoresis (2-DE) and mass spectrometry to identify 26 differentially expressed proteins involved in the metabolism of carbon, nitrogen, and sulphur, as well as in the processes of photosynthesis, protein processing, stress defence, and RNA modification in the albino leaves of ‘White leaf No.1’. Various technologies, including cDNA microarray, amplified fragment length polymorphism (cDNA-AFLP), RNA sequencing, and quantitative real-time PCR (qRT-PCR), have also been used to study genes involved in chlorophyll biosynthesis, chloroplast development, carbon fixation, and biosynthesis of secondary metabolites in ‘White leaf No.1’ (Ma et al., 2012; Xiong et al., 2013; Yuan et al., 2015; Li et al., 2016a). The level of protein succinylation/acetylation in ‘White leaf No.1’ is considered to be related to photosynthesis, carbon fixation, amino acid biosynthesis, and porphyrin and chlorophyll metabolism (Xu et al., 2017a; Xu et al., 2017b).

Similar to the low-temperature dependence of leaf phenotypic changes in the tea cultivar ‘White leaf No.1’, the tea cultivar ‘Xiaoxueya’, which was bred from a natural albino mutant tea plant, produces yellowish to whitish leaves when the environmental temperature is below 20°C in early spring (Du et al., 2006; Du et al., 2008; Ma et al., 2012). Moreover, the cultivar ‘Xiaoxueya’ has even higher levels of amino acids than ‘White leaf No.1’ (Du et al., 2006), prompting an increasing number of tea farmers to grow ‘Xiaoxueya’ for the production of quality green tea. However, the chemical and genetic mechanisms underlying the albino leaf growth of ‘Xiaoxueya’ remain unknown, which has resulted in fluctuations in chemical compositions and tea quality. Overall, an understanding of the chemical and genetic mechanisms leading to ‘Xiaoxueya’ albinism will help improve the production of quality tea. Accordingly, changes in chemical compositions and expression of genes involved in biosynthetic pathways related to pigments, caffeine, theanine, and catechins during the development of albino leaves in the cultivar ‘Xiaoxueya’ were investigated in the present study.

## Materials and Methods

### Plant Materials

Six-year-old tea plants of the cultivar ‘Xiaoxueya’ grown in the field at Daqing Tea Farm (Hangzhou, China; latitude: 30.05°N, longitude: 119.87°E) were used in this study. When the tea shoots developed to the stage with four/five leaves and a bud, apical buds and first (1st), second (2nd), and third (3rd) leaves were sampled, which occurred on May 16, 2014 (at 11:00 a.m.; max. temperature: 27°C; min. temperature: 19°C). Ten-year-old plants of ‘Longjing 43’, a normal tea cultivar widely planted in Zhejiang Province, were cultivated in the field at the Tea Research Institute of the Chinese Academy of Agricultural Sciences (Hangzhou, China; latitude: 30.17°N, longitude: 120.08°E). The apical buds and 1st, 2nd, and 3rd leaves were sampled when the shoots of ‘Longjing 43’ grew to a similar stage as those of ‘Xiaoxueya’, on April 30, 2016 (at 11:00 a.m.; max. temperature: 29°C; min. temperature: 17°C). The samples were packed into Ziploc bags, immediately frozen in liquid nitrogen and stored at −80°C until use. Three biological replicates were used for each biochemical assay. The two cultivars do not share the same genetic background.

### Determination of Pigment Content

Samples (0.20 g) of ‘Xiaoxueya’ leaves were ground and extracted with 10 ml of pre-cooled acetone and 0.1 g of polyvinylpolypyrrolidone (PVPP) in a mortar, and the extracts were centrifuged at 12,000 rpm and 4°C for 15 min. The supernatants were collected for high-performance liquid chromatography (HPLC) detection of pigment components with an LC-20AT HPLC system (Shimadzu, Kyoto, Japan). The HPLC conditions were as follows: 20 µl injection volume; TC-C18 5 µm, 4.6 × 150 mm column (Agilent Technologies Inc., Santa Clara, USA); 35°C oven temperature; acetonitrile/acetic acid/water (6/1/193, v/v/v) for mobile phase A, acetonitrile/methanol/chloroform (15/4/1, v/v/v) for mobile phase B; 1 ml/min flow rate; and 440 nm detection wavelength. The gradient elution conditions were as follows: a linear gradient increasing from 80% to 100% mobile phase B over 20 min, 100% mobile phase B for an additional 15 min, a linear gradient decreasing from 100% to 80% mobile phase B over 5 min, and then 80% mobile phase B for an additional 5 min. Using an external standard-based method (Li et al., 2016c), the examined pigments, consisting of chlorophyll *a*, chlorophyll *b*, neoxanthin, violaxanthin, lutein, and β-carotene, were quantified *via* comparison with authentic reference compounds.

### Transmission Electron Microscopy (TEM) Analysis

The chloroplast ultrastructure of ‘Xiaoxueya’ leaves was investigated *via* TEM according to a previously described method (Li et al., 2016c). Tissue samples were cut into small pieces and fixed overnight with 2.5% (v/v) glutaraldehyde in 0.1 M phosphate buffer (pH 7.0) at 4°C. The pre-fixed samples were then washed three times with 0.1 M phosphate buffer (pH 7.0), re-fixed for 2 h with 1% (v/v) OsO_4_ in 0.1 M phosphate buffer (pH 7.0) and washed three times using 0.1 M phosphate buffer (pH 7.0) as before. The fixed samples were dehydrated using a series of ethanol solutions (30, 50, 70, 80, 90, 95, and 100%, v/v) for 20 min each and dehydrated in pure acetone for 20 min. The dehydrated samples were placed in a mixture of acetone and epoxy resin (1:1, v/v) for 1 h at room temperature, in a mixture of acetone and epoxy resin (1:3, v/v) for 3 h, and in pure epoxy resin overnight. Ultrathin sections were cut using an EM UC7 ultratome (Leica, Schott, Germany). The sections were stained with alkaline lead citrate and uranyl acetate for 5–10 min and observed by TEM using an H-7650 (Hitachi Ltd., Tokyo, Japan).

### Measurement of Alkaloids, Catechins, Total Polyphenols, and Amino Acids

Leaf samples (0.25 g) of ‘Xiaoxueya’ were ground and extracted with 10 ml of ethanol/water (3/1, v/v) for 10 min. The supernatants were collected for chemical composition determination after the extracts were centrifuged at 4°C and 12,000 rpm for 15 min. The concentrations of alkaloids and catechins were determined by HPLC according to methods by Dong et al. (2011). According to methods previously reported in the literature (Liang et al., 2008), the total polyphenol and total amino acid contents were determined by spectrophotometry with ferrous tartrate and ninhydrin colourimetry, respectively. The amounts of major amino acids were analysed by HPLC coupled with pre-column derivatization with 2,4-dinitrofluorobenzene (Li et al., 2016b).

### Total RNA Isolation and qRT-PCR Analysis

Total RNA was isolated from tissues of ‘Xiaoxueya’ and ‘Longjing 43’ using the RNAprep Pure Plant Kit (Tiangen, Beijing, China) following the manufacturer’s instructions. The extracted RNA samples were examined by electrophoresis on a 1.5% agarose gel, and absorbance was measured at 260 and 280 nm using a NanoDrop ND-2000 spectrophotometer (Thermo Fisher Scientific, Inc., USA) to assess RNA integrity, concentration, and purity. SuperScript® III First-Strand Synthesis System (Invitrogen, CA, USA) and oligo(dT)_20_ primer were used to synthesize first-strand cDNA.

The qRT-PCR primers for testing gene expression were designed using Primer-BLAST (http://www.ncbi.nlm.nih.gov/tools/primer-blast/) based on sequences obtained from unigenes of the tea transcriptome (SRA061043, Wang et al., 2013) and the nucleotide database of National Center for Biotechnology Information (NCBI). The accession numbers of gene sequences, primer sequences, and corresponding amplicon lengths are shown in **Table S1**. The *β-actin* gene was chosen as a reference gene to normalize the amount of cDNA in different samples. The reagent SYBR® *Premix Ex Taq*™ II (TaKaRa Biotechnology Co., Ltd., Dalian, China) and an ABI 7500 real-time PCR instrument (Applied Biosystems Co., Ltd., USA) were used to carry out the reactions. Three independent biological replicates were used for each gene expression test. The relative expression levels of genes were calculated following the method (Livak and Schmittgen, 2001).

### Subcellular Localization of the DXS1, DXS2, POR1, and POR2 Proteins

Open reading frame sequences of genes *DXS1*, *DXS2*, *POR1*, and *POR2* were amplified without stop codons *via* reverse transcription PCR (RT-PCR) using KOD-Plus-Neo DNA polymerase (TOYOBO Co., Ltd., Japan) and the primers listed in **Table S2**. The amplified fragments were digested using the restriction enzymes BamHI and SalI (Thermo Fisher Scientific, USA) and ligated into the vector 35S-sGFP, which contains a C-terminal sGFP fragment, using T4 DNA ligase (New England Biolabs Inc., USA). Rice protoplasts were isolated and transformed with the constructed vectors, as previously reported (Wang et al., 2015). A Nikon C2-ER confocal laser scanning microscope was used for capturing images.

### Statistical Analysis

**Figure 1:**
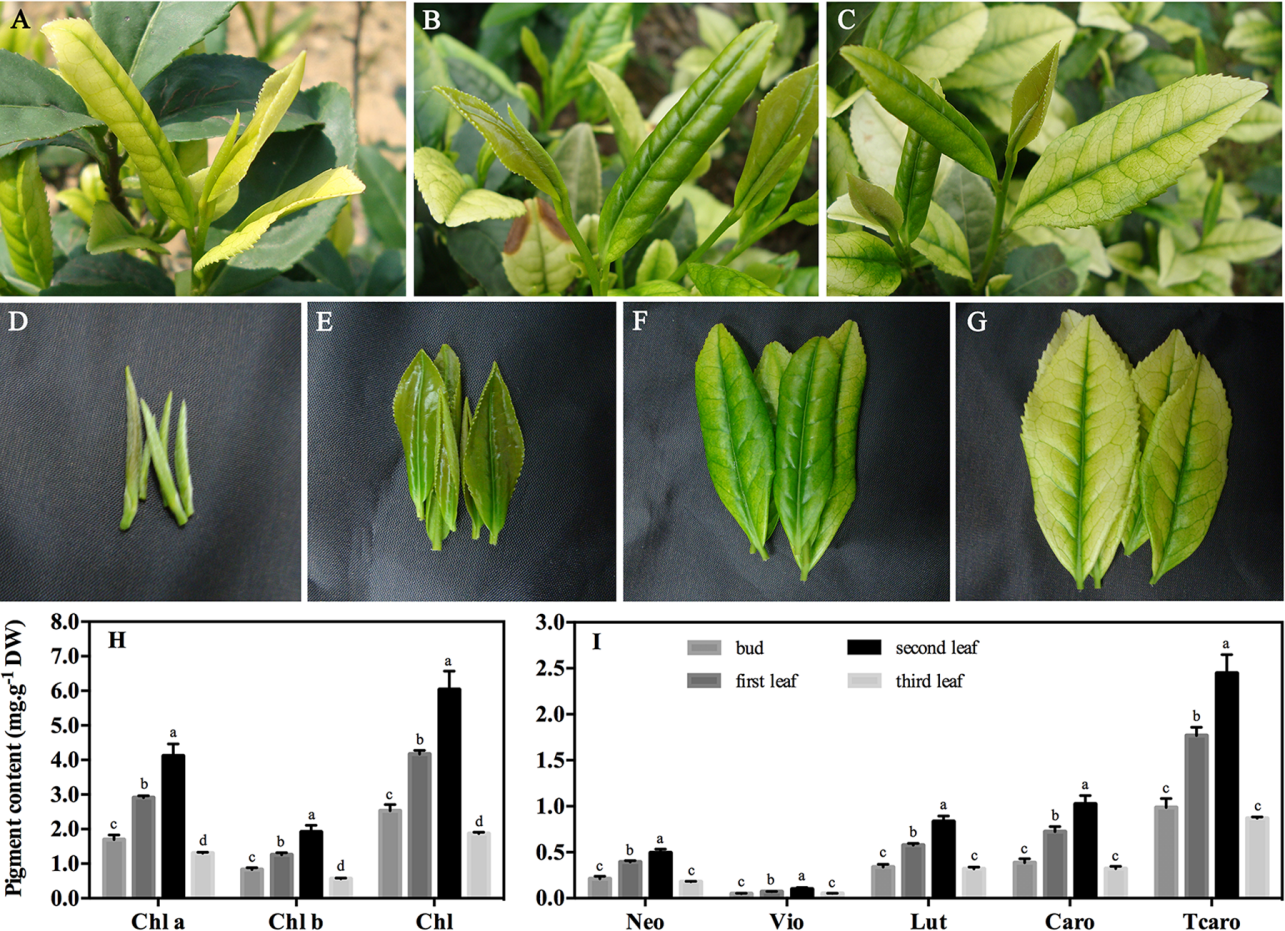
Phenotypic characteristics and pigment content of ‘Xiaoxueya’ leaves. **(A)** New shoots with two leaves and a bud photographed on April 15, 2014. **(B)** New shoots with two leaves and a bud photographed on May 16, 2014. **(C)** New shoots with three leaves and a bud photographed on May 16, 2014. **(D**–**G)** Buds and first, second, and third leaves photographed on May 16, 2014. **(H)** Chlorophyll and **(I)** carotenoid contents in different leaves. DW, dry weight. Chl a, chlorophyll a. Chl b, chlorophyll b. Chl, total chlorophylls. Neo, neoxanthin. Vio, violaxanthin. Lut, lutein. Caro, β-carotene. Tcaro, total carotenoids. Data are presented as the mean of three replicates (± standard deviation). The values in columns labelled with different letters are significantly different (*p* value ≤ 0.05).

All data are presented as the mean ± standard deviation. Significant differences among data were analysed by one-way analysis of variance (ANOVA) followed by Duncan’s multiple range test (**Figure 1**, **Table 1**) or Student’s *t* test (**Figures 3**–**5**, **Figures S1** and **S2**) using SPSS 20.0 software (IBM Corporation, USA). For the metabolite data in **Figure 1** and **Table 1**, *p* values ≤ 0.05 were considered to indicate statistically significant differences. For the gene expression data in **Figures 3**–**5**, **Figures S1** and **S2**, a *p* value ≤ 0.05 and an absolute value of log2FoldChange ≥ 1 were regarded as indicators of significant differential expression.

**Table 1:** Contents of metabolites in ‘Xiaoxueya’ leaves (mg/g DW).

Metabolite	Bud	First leaf	Second leaf	Third leaf
Theobromine	4.38 ± 0.60 a	2.81 ± 0.08 b	1.80 ± 0.08 c	0.61 ± 0.04 d
Caffeine	32.78 ± 0.27 c	38.69 ± 0.78 a	34.29 ± 0.94 b	24.34 ± 0.60 d
Total alkaloids	37.16 ± 0.88 b	41.49 ± 0.85 a	36.09 ± 1.02 b	24.95 ± 0.56 c
Gallic acid	1.07 ± 0.14 a	1.17 ± 0.07 a	0.83 ± 0.05 b	0.25 ± 0.01 c
EC	2.58 ± 0.20 d	6.12 ± 0.36 c	9.38 ± 0.28 a	7.85 ± 0.31 b
C	2.44 ± 0.01 a	1.85 ± 0.09 b	1.86 ± 0.03 b	1.92 ± 0.02 b
EGC	5.49 ± 0.99 c	16.34 ± 2.47 b	27.33 ± 1.19 a	27.38 ± 0.62 a
GC	2.41 ± 0.06 b	3.28 ± 0.25 a	3.42 ± 0.11 a	2.00 ± 0.28 b
EGCG	75.41 ± 3.34 c	103.76 ± 5.71 a	89.55 ± 2.90 b	47.13 ± 2.01 d
ECG	24.24 ± 0.88 b	27.06 ± 0.99 a	19.79 ± 1.11 c	8.44 ± 0.35 d
Total catechins	112.58 ± 5.36 b	158.42 ± 9.58 a	151.34 ± 5.11 a	94.71 ± 3.03 c
Total polyphenols	277.30 ± 7.71 b	332.80 ± 17.73 a	283.04 ± 9.26 b	162.16 ± 4.91 c
Aspartic acid	3.13 ± 0.10 bc	2.32 ± 0.21 c	3.24 ± 0.09 b	7.63 ± 0.72 a
Glutamic acid	4.89 ± 0.13 b	3.21 ± 0.30 d	3.89 ± 0.13 c	7.90 ± 0.41 a
Serine	0.88 ± 0.02 bc	0.76 ± 0.06 c	0.95 ± 0.02 b	1.96 ± 0.15 a
Theanine	8.68 ± 0.43 b	3.59 ± 0.15 c	8.40 ± 0.18 b	23.38 ± 1.45 a
Alanine	0.31 ± 0.00 b	0.18 ± 0.01 c	0.37 ± 0.03 b	1.03 ± 0.06 a
γ-Aminobutyric acid	0.83 ± 0.03 a	0.53 ± 0.03 c	0.62 ± 0.09 bc	0.70 ± 0.02 b
Total amino acids	41.94 ± 0.14 b	32.66 ± 1.22 c	38.74 ± 0.55 b	67.43 ± 4.02 a
Ratio of polyphenols to amino acids	6.61 ± 0.21 c	10.19 ± 0.28 a	7.31 ± 0.15 b	2.41 ± 0.20 d

Data are presented as the mean of three replicates (± standard deviation). Data marked with different letters indicate statistically significant differences among samples (p value ≤ 0.05). DW, dry weight. EC, (−)-epicatechin. C, (+)-catechin. EGC, (−)-epigallocatechin. GC, (+)-gallocatechin. EGCG, (−)-epigallocatechin-3-O-gallate. ECG, (−)-epicatechin-3-O-gallate.

## Results

### Phenotypic Characteristics and Pigment Content

The cultivar ‘Xiaoxueya’ is a temperature-sensitive albino tea plant, and its leaf colour depends on the environmental temperature (Du et al., 2006; Du et al., 2008). Generally, the young leaves produced when the environmental temperature is below 20°C in early spring are pale yellow or white in colour (**Figures 1A, G**), whereas the new leaves produced when the temperature increases to above 20°C in the late spring become greener in colour (**Figures 1B–F**). These confirmed that the albino phenotype of cultivar ‘Xiaoxueya’ depends on the environmental temperature at which it grows.

Pigment analysis showed that the contents of chlorophyll *a*, chlorophyll *b*, total chlorophylls, and carotenoids in the 3rd albino leaf under the apical bud, which grew in early April when the temperature was below 20°C, were significantly lower than those in the 1st and 2nd leaves which grew in May when the temperature was above 20°C (*p* value < 0.05; **Figures 1H, I**). These indicate that a deficiency in chlorophylls and carotenoids is responsible for the albino phenotype of tea cultivar ‘Xiaoxueya’.

### Chloroplast Ultrastructure

**Figure 2:**
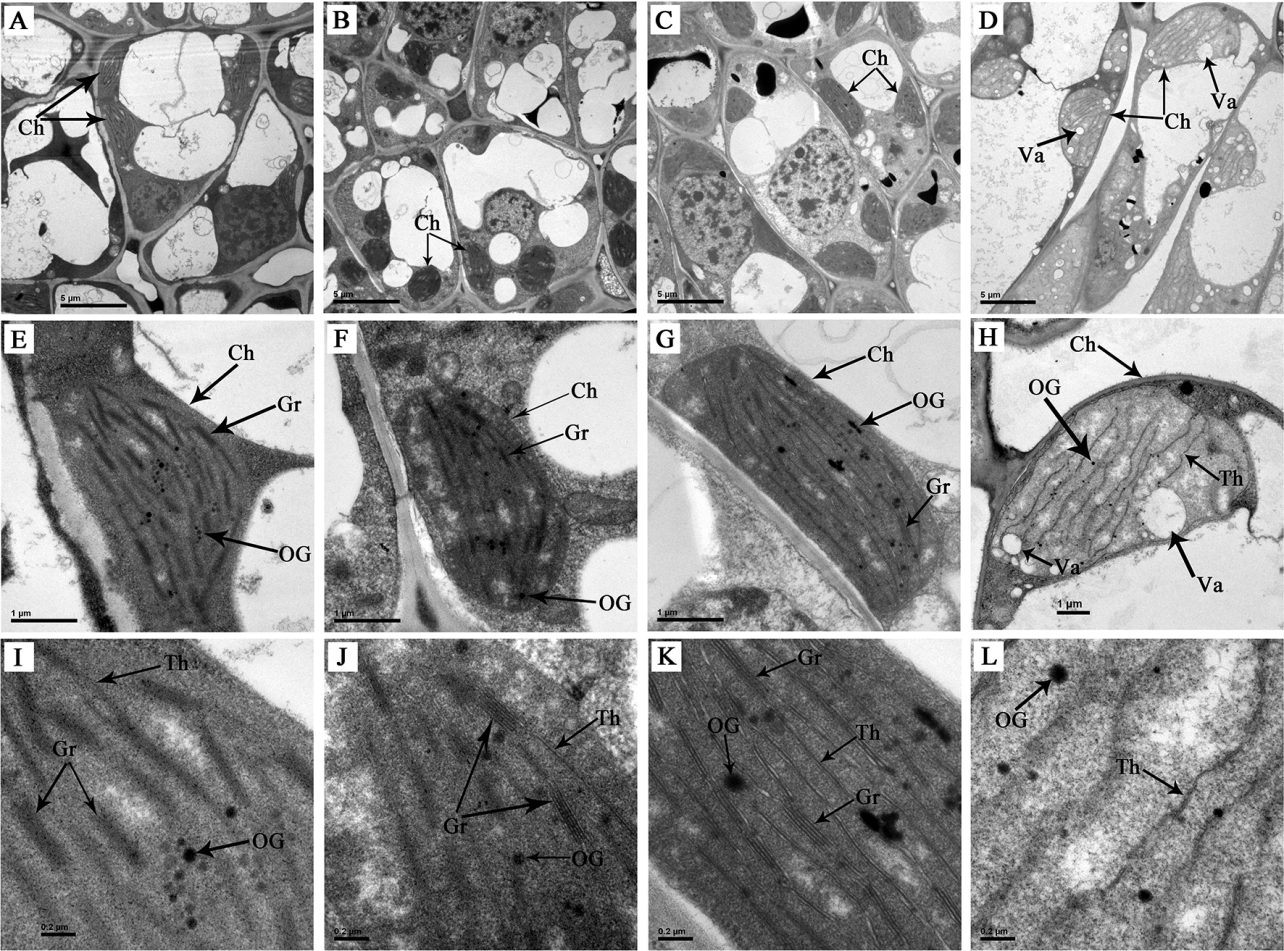
Chloroplast ultrastructure of ‘Xiaoxueya’ leaves. **(A**, **E**, **I)** Bud. **(B**, **F**, **J)** First leaf. **(C**, **G**, **K)** Second leaf. **(D**, **H**, **L)** Third leaf. Ch, chloroplast. OG, osmiophilic granule. Gr, grana. Th, thylakoid. Va, vacuole.

The chloroplast, a type of organelle known as a plastid, is characterized by a high concentration of pigments and is responsible for leaf colour and photosynthesis. Observation on ultrastructure of the chloroplasts in differently coloured leaves of ‘Xiaoxueya’ showed that the green bud (**Figures 2A, E, I**), 1st leaf (**Figures 2B, F, J**), and 2nd leaf (**Figures 2C, G, K**), which grew in May exhibited a well-established chloroplast ultrastructure consisting of abundant thylakoids and tightly stacked grana. However, the 3rd leaf, which grew in the early April and lacked chlorophylls, exhibited an abnormal internal chloroplast morphology (**Figures 2D, H, L**), with many vacuoles but few thylakoids and no granum stacking observed. These suggest that chloroplast developmental disorders with a decrease in the number of thylakoids and deficiency in grana lamellae are important factors leading to the albino phenotype of ‘Xiaoxueya’.

### Contents of Foliar Alkaloids, Catechins, and Amino Acids

To explore the relationship of the albino phenotype and foliar chemical compositions relating to tea quality, the contents of alkaloids, catechins, polyphenols, and amino acids in the tested tea leaf samples were determined. The results showed that the albino leaf (3rd leaf) had significantly lower contents of theobromine, caffeine, and total alkaloids than the green organs did (bud, 1st and 2nd leaves; *p* value < 0.001; **Table 1**). The concentrations of gallic acid and galloylated catechins, including (−)-epigallocatechin-3-*O*-gallate (EGCG) and (−)-epicatechin-3-*O*-gallate (ECG), were also significantly lower in the albino 3rd leaf than in the green bud, 1st and 2nd leaves (*p* value < 0.001). However, there were no definite trends for the changes in non-galloylated catechins, including (−)-epicatechin (EC), (+)-catechin (C), (−)-epigallocatechin (EGC), and (+)-gallocatechin (GC), between the albino and the green leaves. The contents of total catechin and total polyphenol in the albino leaf were lower than those in the green bud, 1st leaf, and 2nd leaf (*p* value < 0.05). The contents of amino acids, including aspartic acid, glutamic acid, serine, theanine, and alanine, were significantly higher in the albino leaf than in green leaves (*p* value < 0.001), with the exception of γ-aminobutyric acid. A low ratio of polyphenols to amino acids (P/A) is considered to be a good indicator of quality green tea, and the P/A of the albino leaf was markedly lower than that of the green bud and green leaves (*p* value < 0.001; **Table 1**), suggesting that the albino leaf of the tea cultivar ‘Xiaoxueya’ is a quality material for processing green tea.

### Expression of Genes Involved in Photosynthetic Pigment Biosynthesis

**Figure 3:**
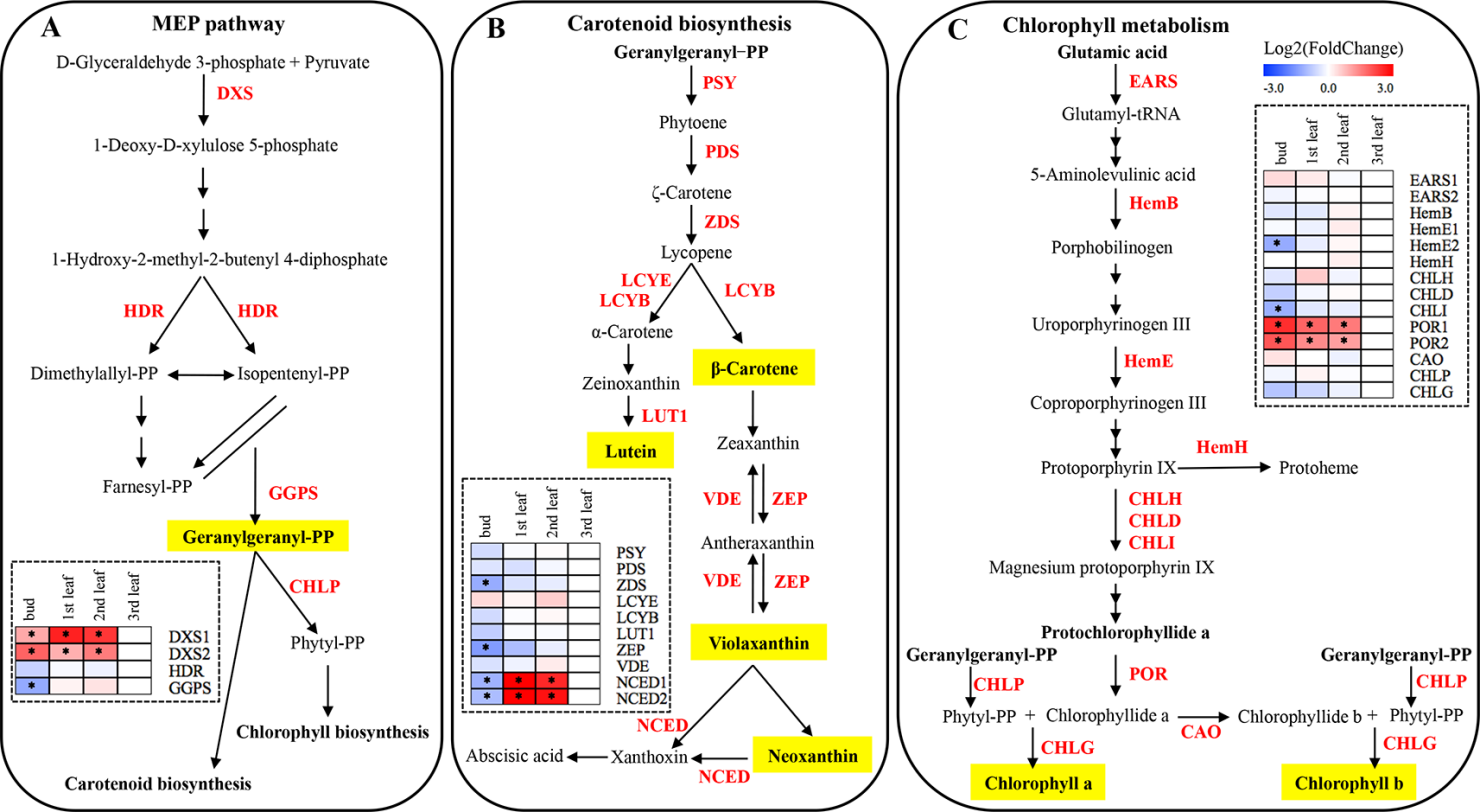
MEP, carotenoid and chlorophyll biosynthetic pathways in plants, and expression patterns of genes related to pigment metabolism in ‘Xiaoxueya’ leaves. **(A)** Genes involved in the MEP biosynthetic pathway. **(B)** Genes involved in the carotenoid biosynthetic pathway. **(C)** Genes involved in the chlorophyll biosynthetic pathway. Genes used for expression analysis are shown in red. The heat maps were generated using the mean of three replicates and MeV software. The blue-white-red schemes are labelled above the heat map. Red and blue represent relatively high and low expression levels, respectively, compared to those shown in white. Asterisks (*) indicate significant differences (*p* value ≤ 0.05) and |log2FoldChange| ≥ 1 between the values from the sample and third leaf.

The 2-C-methyl-D-erythritol-4-phosphate (MEP) pathway is important for the generation of photosynthetic pigments such as chlorophylls and carotenoids. The qRT-PCR tests showed that the expression of the gene encoding 1-deoxy-D-xylulose-5-phosphate synthase (*DXS1* and *DXS2*) was significantly lower in the albino leaf than in the green bud and leaf of ‘Xiaoxueya’ (*p* value < 0.01; **Figure 3A** and **Figure S1A**). There was no significant difference in the expression level of the 4-hydroxy-3-methylbut-2-en-1-yl diphosphate reductase gene (*HDR*) among the four samples of ‘Xiaoxueya’ (**Figure 3A** and **Figure S1A**). The expression of the geranylgeranyl diphosphate synthase gene (*GGPS*) in the albino leaf was significantly higher than that in the bud (*p* value < 0.01; **Figure 3A** and **Figure S1A**). To exclude the effects of leaf maturity on the gene expression, we further determined the expression patterns of *DXS1* and *DXS2* in the normal green cultivar ‘Longjing 43’ and found no significant difference in *DXS1* expression level among the tested samples (**Figure 5**), yet expression of *DXS2* was significantly higher in the 3rd leaf than in the bud and 1st leaf of the cultivar ‘Longjing 43’ (*p* value < 0.001; **Figure 5**). These findings demonstrate that the downregulated expression of *DXS1* and *DXS2* is responsible for the lack of foliar pigments in the albino leaves of ‘Xiaoxueya’.

There was no significant difference between the tested samples of the cultivar ‘Xiaoxueya’ in expression of the genes encoding phytoene synthase (PSY), phytoene desaturase (PDS), lycopene ε-cyclase (LCYE), lycopene β-cyclase (LCYB), carotene ε-monooxygenase (LUT1), and violaxanthin de-epoxidase (VDE) (**Figure 3B** and **Figure S1B**). Interestingly, the transcript abundances of genes encoding ζ-carotene desaturase (ZDS) and zeaxanthin epoxidase (ZEP) in the albino leaf were 2-fold higher than those in the green bud (*p* value < 0.01; **Figure 3B** and **Figure S1B**). The 9-cis-epoxycarotenoid dioxygenase (NCED) is an enzyme to catalyse the cleaving of violaxanthin and neoxanthin to form xanthoxin. The expression of *NCED1* and *NCED2* in the albino leaf of ‘Xiaoxueya’ was significantly higher than that in the bud (*p* value < 0.01) but significantly lower than that in green 1st and 2nd leaves (*p* value < 0.001; **Figure 3B** and **Figure S1B**). Conversely, *NCED1* and *NCED2* expression in ‘Longjing 43’ leaves displayed no trend similar to that observed for ‘Xiaoxueya’ (**Figure 5**). Based on these results and the above analysis of genes involved in the MEP pathway, it is considered that the inhibition of the expression of *DXS1* and *DXS2* would be key for the reduction in carotenoid levels in ‘Xiaoxueya’ albino leaves.

Fourteen genes involved in the chlorophyll biosynthetic pathway were identified, and their expression levels were compared among the four tested samples of the cultivar ‘Xiaoxueya’. The expression levels of genes encoding glutamate-tRNA ligase (EARS1, EARS2), δ-aminolevulinic acid dehydratase (HemB), uroporphyrinogen decarboxylase (HemE1), ferrochelatase (HemH), magnesium chelatase subunit H and D (CHLH, CHLD), chlorophyllide a oxygenase (CAO), geranylgeranyl diphosphate reductase (CHLP), and chlorophyll synthase (CHLG) showed no significant differences (**Figure 3C** and **Figure S1C**), though the transcriptional levels of HemE2 and magnesium chelatase subunit I (CHLI) in the ‘Xiaoxueya’ albino leaf were significantly higher than those in the bud (*p* value < 0.01; **Figure 3C** and **Figure S1C**). The expression of two members of gene encoding protochlorophyllide oxidoreductase (*POR1* and *POR2*) was significantly downregulated in the albino leaf, compared to the green bud and leaf (*p* value < 0.001; **Figure 3C** and **Figure S1C**). Assessment of the normal green leaf cultivar ‘Longjing 43’ showed that leaf maturity had no significant effect on the expression of *POR1* and *POR2* (**Figure 5**). Based on these results, it is considered that the suppressed expression of *POR1* and *POR2*, along with low expression of *DXS1* and *DXS2*, might be responsible for the decreased accumulation of chlorophyll *a* and chlorophyll *b* in albino leaf of ‘Xiaoxueya’.

### Expression of Genes Involved in the Biosynthesis of Quality-Related Metabolites

**Figure 4:**
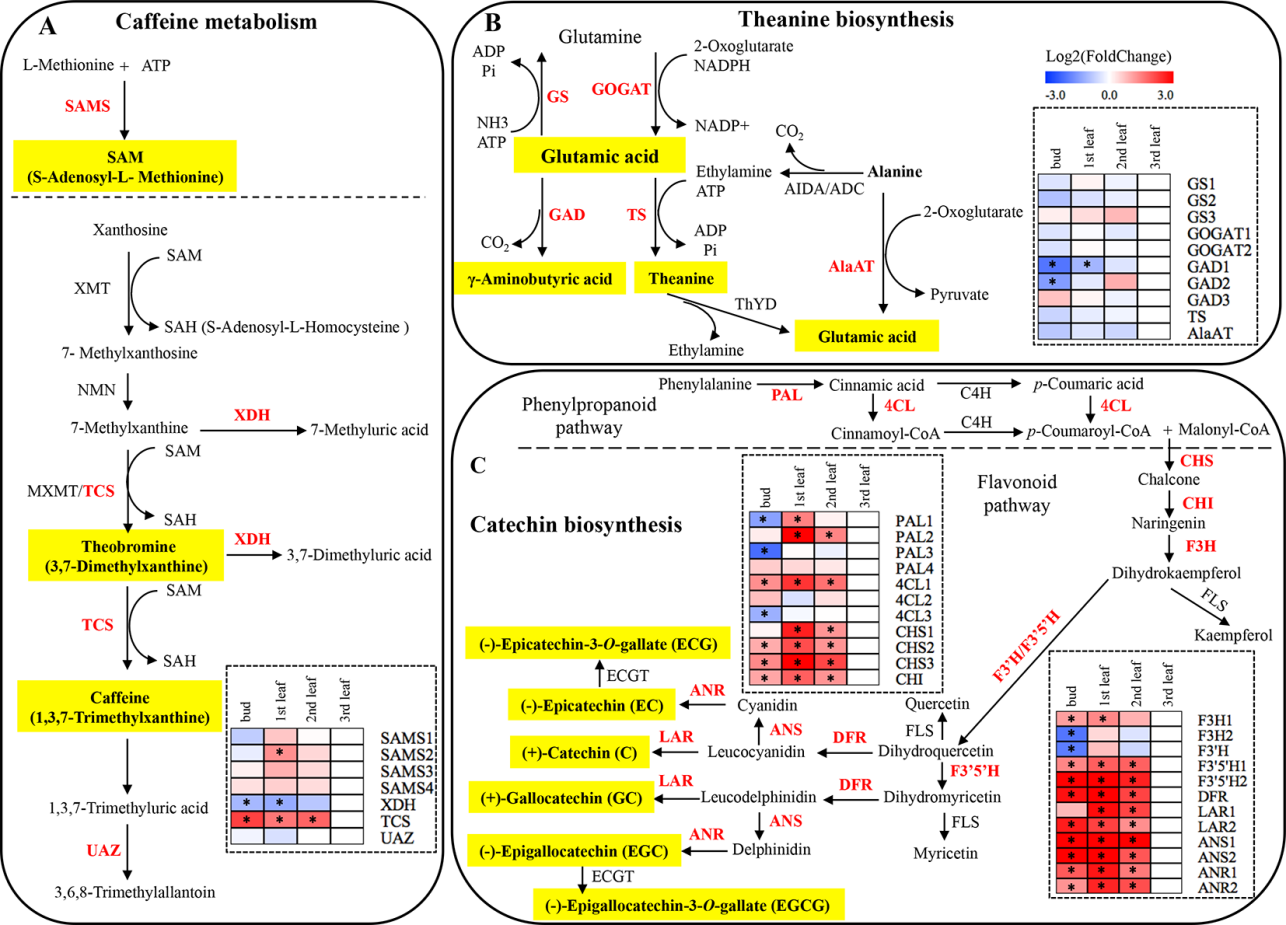
Caffeine, theanine and catechin biosynthetic pathways in plants, and expression patterns of genes related to quality metabolites in ‘Xiaoxueya’ leaves. **(A)** Genes involved in the caffeine metabolic pathway. **(B)** Genes involved in the theanine biosynthetic pathway. **(C)** Genes involved in the catechin biosynthetic pathway. Genes used for expression analysis are shown in red. The heat maps were generated using the mean of three replicates and MeV software. The blue-white-red schemes are labelled above the heat map. Red and blue represent relatively high and low expression levels, respectively, compared to those shown in white. Asterisks (*) indicate significant differences (*p* value ≤ 0.05) and |log2FoldChange| ≥ 1 between the values from the sample and third leaf.

**Figure 5:**
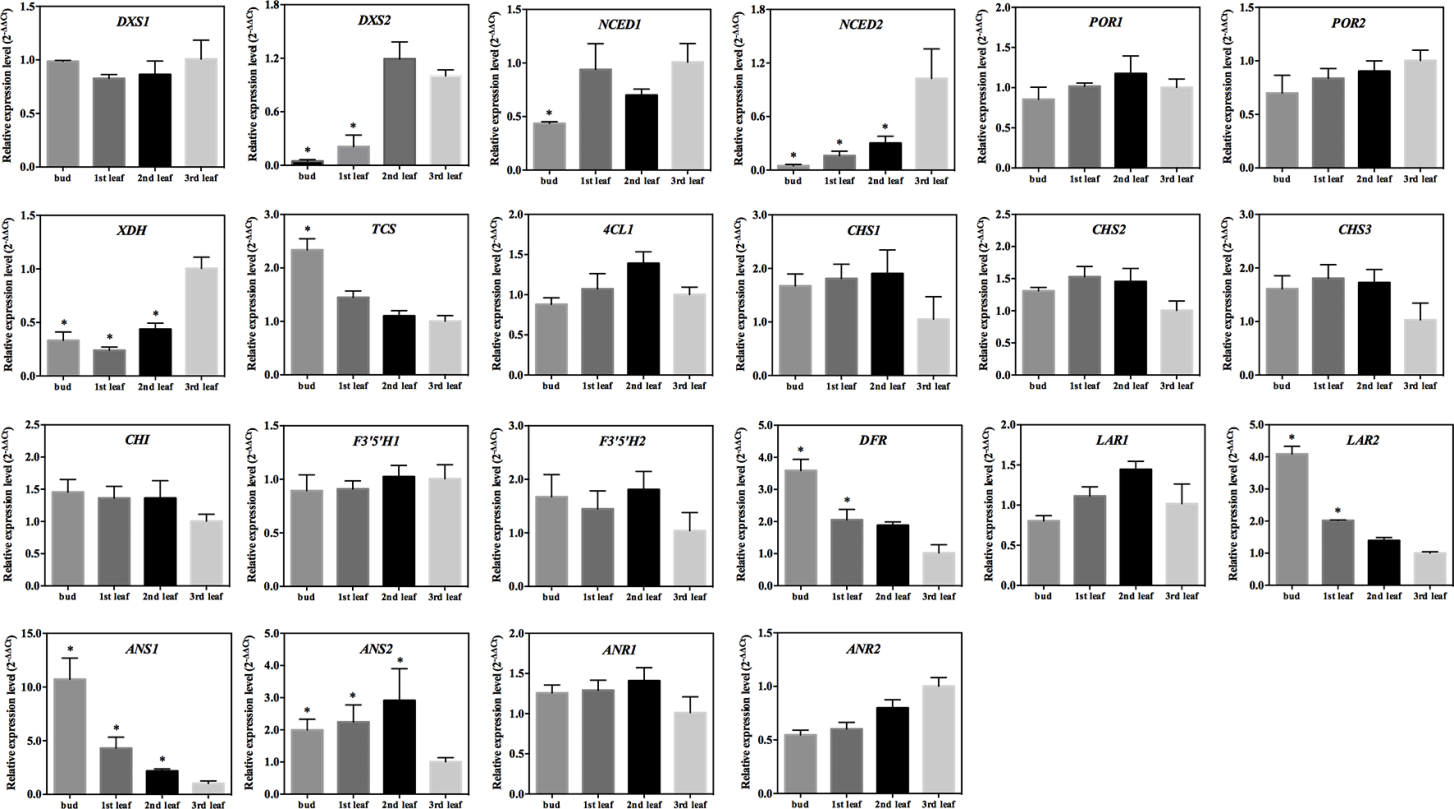
Expression patterns of 22 genes in ‘Longjing 43’ leaves. Data are presented as the mean of three replicates (± standard deviation). Columns marked with asterisks indicate statistically significant differences (*p* value ≤ 0.05) and |log2FoldChange| ≥ 1 between values from the sample and third leaf.

S-Adenosylmethionine synthetase (SAMS) catalyses the synthesis of S-adenosyl-L-methionine (SAM). The expression levels of four members of the SAMS gene family (*SAMS1*, *SAMS2*, *SAMS3*, *SAMS4*) were investigated in the tested samples of ‘Xiaoxueya’, among which the expression of *SAMS2* was significantly downregulated in the albino leaf, compared to the 1st leaf (*p* value < 0.001); in contrast, no significant differences in expression levels of *SAMS1*, *SAMS3*, and *SAMS4* were detected between the tested samples (**Figure 4A** and **Figure S2A**). Additionally, the expression of the gene encoding xanthine oxidase (XDH) in the albino leaf was significantly upregulated, compared to the bud and 1st leaf (*p* value < 0.01) (**Figure 4A** and **Figure S2A**). The transcriptional level of the tea caffeine synthase gene (*TCS*) was significantly lower in the ‘Xiaoxueya’ albino leaf than in all green tissues (*p* value < 0.001; **Figure 4A** and **Figure S2A**), but there was no significant difference in urate oxidase gene (*UAZ*) expression among the tested samples of ‘Xiaoxueya’ (**Figure 4A** and **Figure S2A**). Analysis on the samples of cultivar ‘Longjing 43’ showed that expression of *XDH* was significantly upregulated in the 3rd leaf, compared to the bud and 1st leaf (*p* value < 0.01; **Figure 5**), displaying a similar trend as the cultivar ‘Xiaoxueya’. TCS transcription in the 3rd leaf of ‘Longjing 43’ was 2.3-fold lower than that in the bud but not significantly different from that in the 1st or 2nd leaf (**Figure 5**). These results suggest that the low expression of *TCS* could be a key factor in the reduced alkaloid levels in the albino leaf of ‘Xiaoxueya’.

Glutamine synthetase (GS) catalyses the biosynthesis of glutamine using glutamic acid, ammonia, and ATP as substrates. Conversely, glutamic acid can be produced from glutamine, 2-oxoglutarate, and reduced nicotinamide adenine dinucleotide phosphate (NADPH) by glutamate synthase (GOGAT); alanine transaminase (AlaAT) also catalyses the synthesis of glutamic acid using alanine and 2-oxoglutarate as substrates. The intermediate ethylamine in theanine biosynthesis pathway is transformed from alanine by alanine decarboxylase (AIDA), and the ethylamine is subsequently used to synthesize theanine by theanine synthetase (TS) in the presence of glutamic acid. Unexpectedly, the transcriptional levels of the genes *GS1*, *GS2*, *GS3*, *GOGAT1*, *GOGAT2*, *TS*, and *AlaAT* in ‘Xiaoxueya’ albino leaf were not significantly different from those in other leaf or bud (**Figure 4B** and **Figure S2B**). γ-Aminobutyric acid is formed through decarboxylation of glutamic acid by glutamate decarboxylase (GAD), and the expression of *GAD1* and *GAD2* in the albino leaf was significantly higher than that in the bud (*p* value < 0.01; **Figure 4B** and **Figure S2B**). These findings indicate that high levels of amino acids, including theanine, in the pigment-deficient leaves of ‘Xiaoxueya’ might not be resulted from differences in mRNA levels of the key genes involved in theanine biosynthesis.

The expression of 23 genes involved in phenylpropanoid and flavonoid biosynthesis was examined so as to explore the reason for the low levels of catechins and polyphenols in ‘Xiaoxueya’ albino leaves. Expression of two members of the gene encoding phenylalanine ammonia-lyase (*PAL1*, *PAL3*) was significantly upregulated in the ‘Xiaoxueya’ albino leaf compared to the bud, but *PAL2* was significantly downregulated in the albino leaf compared to the 1st and 2nd leaves (*p* value < 0.001; **Figure 4C** and **Figure S2C**). In addition, the expression of 4-coumarate-CoA ligase gene (*4CL1*) in all of the tested green bud and leaves of ‘Xiaoxueya’ was significantly upregulated compared to the albino leaf (*p* value < 0.01; **Figure 4C** and **Figure S2C**). Expression of the chalcone synthase gene (*CHS1*) in the ‘Xiaoxueya’ albino leaf was significantly downregulated compared to the 1st and 2nd leaves. Furthermore, the expression of *CHS2*, *CHS3*, and the chalcone isomerase gene (*CHI*) in the albino leaf was significantly downregulated compared to green bud and leaves of ‘Xiaoxueya’ (*p* value < 0.01; **Figure 4C** and **Figure S2C**). There was no significant difference in the expression of *4CL1*, *CHS1*, *CHS2*, *CHS3*, and *CHI* in the tested samples of ‘Longjing 43’ (**Figure 5**). The expression of the flavanone 3-hydroxylase gene (*F3H1*) was significantly downregulated in the albino leaf, compared to the green bud and 1st leaf of ‘Xiaoxueya’. However, the expression of *F3H2* was significantly upregulated in the albino leaf, compared to the green bud (*p* value < 0.01; **Figure 4C** and **Figure S2D**). A flavonoid 3’-hydroxylase gene (*F3’H*) exhibited the same expression pattern as *F3H2* in ‘Xiaoxueya’ leaves (**Figure 4C**). Notably, the expression of genes encoding flavonoid 3’,5’-hydroxylase (F3’5’H1, F3’5’H2), dihydroflavonol 4-reductase (DFR), leucoanthocyanidin reductase (LAR2), anthocyanidin synthase (ANS1, ANS2), and anthocyanidin reductase (ANR1, ANR2) was significantly downregulated in the albino leaf compared to the green bud and leaves of ‘Xiaoxueya’ (*p* value < 0.01; **Figure 4C** and **Figure S2D**). In the green leaves of normal cultivar ‘Longjing 43’, expression of *DFR*, *LAR2*, *ANS1*, and *ANS2* in the 3rd leaf was significantly downregulated compared to the bud and 1st leaf (*p* value < 0.05; **Figure 5**), suggesting that the low expression levels of these genes in the ‘Xiaoxueya’ albino leaf depend on the maturity of the leaf, instead of on the albino phenotype. To summarize, specifically suppressed expression of *CHS1*, *CHS2*, *CHS3*, *CHI*, *F3’5’H1*, *F3’5’H2*, *ANR1*, and *ANR2* is considered to be responsible for the low levels of catechins in the albino leaves of the cultivar ‘Xiaoxueya’.

### Subcellular Localization of DXS1, DXS2, POR1, and POR2

**Figure 6:**
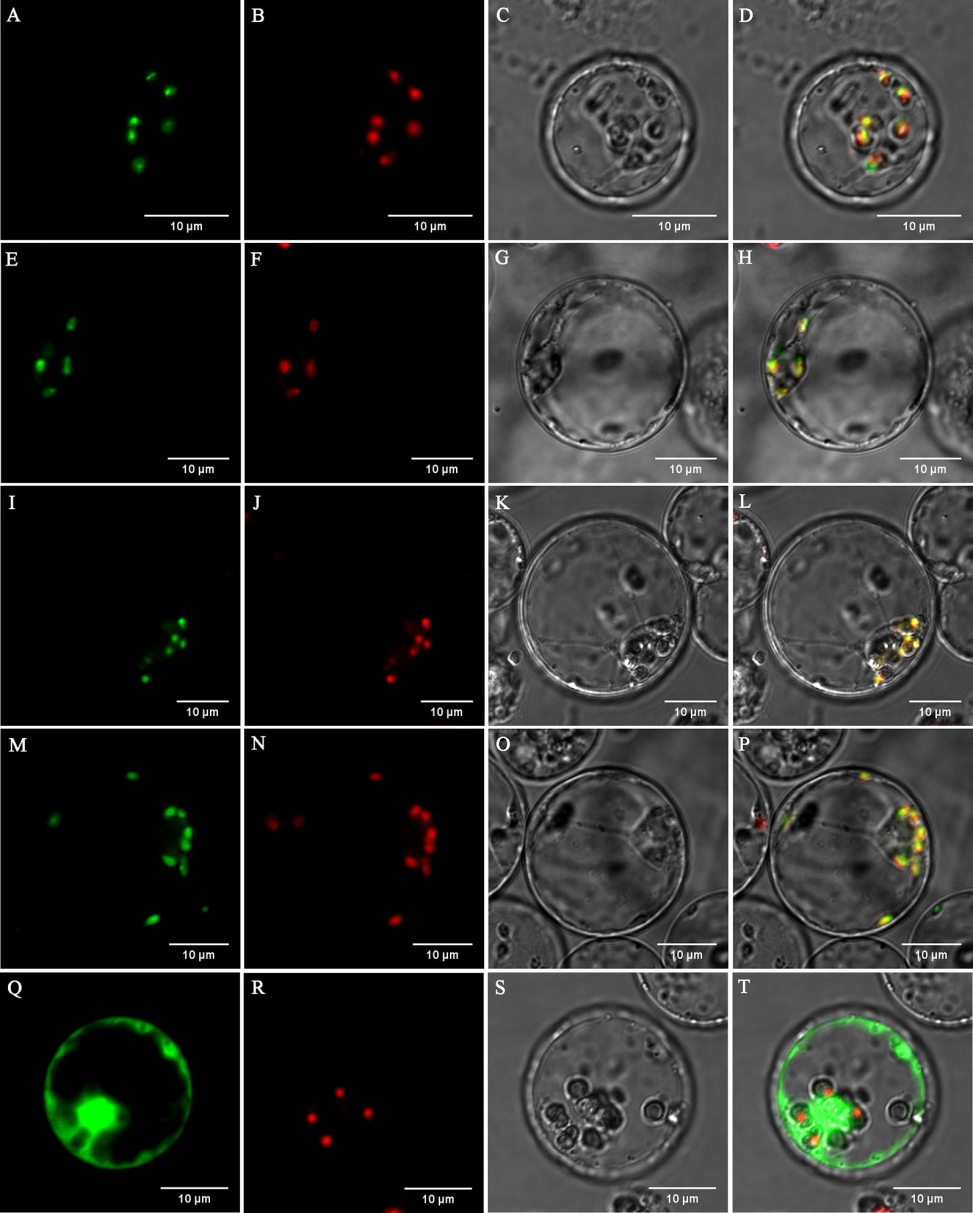
Subcellular localization of DXS1, DXS2, POR1, and POR2 proteins from tea plants in rice cell protoplasts. **(A**–**D)** 35S:DXS1:sGFP, **(E**–**H)** 35S:DXS2:sGFP, **(I**–**L)** 35S:POR1:sGFP, **(M**–**P)** 35S:POR2:sGFP, and **(Q**–**T)** 35S:sGFP were transformed into rice protoplasts. **(A**, **E**, **I**, **M**, **Q)** The green signals indicate GFP fluorescence. **(B**, **F**, **J**, **N**, **R)** The red signals indicate chlorophyll auto-fluorescence. **(C**, **G**, **K**, **O**, **S)** Optical fields. **(D**, **H**, **L**, **P**, **T)** Merged images.

Prediction using the WoLF PSORT tool (http://www.genscript.com/wolf-psort.html) showed that the proteins DXS1, DXS2, POR1, and POR2 are localized to the chloroplast. To verify the localization of these proteins which were significantly suppressed in the ‘Xiaoxueya’ albino leaf at the transcriptional level (**Figure 3** and **Figure S1**), the full-length DXS1, DXS2, POR1, and POR2 proteins were individually fused to GFP and then transiently expressed in rice protoplasts for subcellular localization analysis. The GFP of the control vector emitted green fluorescence throughout the cells (**Figures 6Q, T**), whereas the green fluorescence of DXS1-GFP, DXS2-GFP, POR1-GFP, and POR2-GFP was localized in chloroplasts (**Figures 6A, E, I, M**) and coincided with chlorophyll auto-fluorescence (**Figures 6B, D, F, H, J, L, N, P**). These results confirm that the proteins DXS1, DXS2, POR1, and POR2 are located in chloroplasts.

## Discussion

### Albinism of the Tea Cultivar ‘Xiaoxueya’

The present study on the albino tea cultivar ‘Xiaoxueya’ showed that the levels of chlorophylls and carotenoids in the 3rd albino leaf were lower than those in the green bud, the 1st and 2nd leaves (**Figure 1**). In normal tea cultivars, however, the contents of foliar chlorophylls and carotenoids were increased with leaf maturity in shoots up to the stage of three leaves and a bud (Wang et al., 2012; Samanta et al., 2017). Based on the published literature, and **Figures 2** and **3** in the present study, three factors are considered to be responsible for the reduced contents of chlorophylls and carotenoids. First, the downregulation of DXS1 and DXS2 expression (**Figure 3**) leads to the reduced amount of precursor for biosynthesizing carotenoids and chlorophylls because geranylgeranyl diphosphate, the precursor of carotenoids and chlorophylls, is formed in the plastid MEP pathway initially catalysed by rate-limiting DXS (Estévez et al., 2001). These were confirmed in tomato and *arabidopsis*. Tomato mutant *wls*-2297 which is induced by a 38.6-kb deletion promoted by a single T-DNA insertion affecting the *DXS1* gene, exhibited a severe deficiency in chlorophylls and carotenoids (García-Alcázar et al., 2017). *DXS* over-expression in a transgenic *arabidopsis* led to an increase in chlorophylls and carotenoids (Estévez et al., 2001). Second, the downregulation of *POR1* and *POR2* genes encoding protochlorophyllide oxidoreductase (POR) (**Figure 3**) leads to less accumulation of chlorophyllide *a*. Chlorophyllide *a* is produced by photoreduction of protochlorophyllide *a*, which is mediated by a strictly light-dependent enzyme POR. The chlorophyllide *a* is further esterified and modified to produce chlorophyll *a* and chlorophyll *b* (Griffiths, 1978). Ectopic over-expression of POR gene encoding PORA was confirmed to restore bulk chlorophylls accumulation in a *porB-1 porC-1* double null mutant *arabidopsis* with unstacked thylakoid membranes in chloroplasts and showing a seedling-lethal *xantha* phenotype (Frick et al., 2003; Paddock et al., 2010). POR was downregulated in a light-sensitive albino tea cultivar ‘Baijiguan’ (Wu et al., 2016). Third, aberrant chloroplast development (**Figure 2**) is associated with the defect in pigment biosynthesis. This was confirmed in leaf colour plant mutants. The rice mutants *lta1* and *tcd5* develop albino leaves when grown at temperatures lower than 20°C, accompanying with the lack of well-stacked grana and abundant vacuoles in the abnormal chloroplasts (Peng et al., 2012; Wang et al., 2016).

### Changes in the Chemical Composition of Albino Leaves of the Tea Cultivar ‘Xiaoxueya’

In the normal tea cultivars with green leaves, the content of tea catechins usually increases, while the content of amino acids including theanine decreases with tea leaf maturity (Lin et al., 2003; Song et al., 2012). In contrast, we show here that the albino mature 3rd leaf had higher levels of amino acids but catechins and caffeine amounts were much lower than the less mature 1st and 2nd green leaves.

The less accumulation of tea catechins in the albino 3rd leaf of cultivar ‘Xiaoxueya’ (**Table 1**) is considered to be related to the downregulation of the genes *CHS*, *CHI*, *F3’5’H*, and *ANR* involving in multiple branches of the phenylpropanoid and flavonoid pathways (Wang et al., 2012; Wang et al., 2018). CHS is the first enzyme in the flavonoid pathway, which catalyses the synthesis of chalcone by condensing *p*-coumaroyl-CoA and malonyl-CoA, and then the chalcone is converted by CHI into naringenin, a precursor for downstream metabolites including catechins (Harris et al., 2013; Cheng et al., 2018). CHI-knockdown mutation in *petunia* and *torenia* reduced flavonoid production (Morita et al., 2014). The gene F3’5’H encoding enzyme flavonoid 3’,5’-hydroxylase in tea plant is a crucial synthesis controller of tea catechins including GC, EGC, and EGCG (Wang et al., 2014b). The ANR enzyme converts the anthocyanidins cyanidin and delphinidin into EC and EGC and over-expression of *ANR* increased contents of EC and EGC (Kumar et al., 2013).

The expression of the key genes involved in theanine biosynthesis in the present study showed no significant differences between albino 3rd leaf and green leaves (**Figure 4**) though the albino leaf had higher content of theanine than the later. This remains to be further investigated.

The reduced theobromine and caffeine in the albino leaf (**Table 1**) is considered to be related to its lower transcription of *TCS* (**Figure 4**), compared to green leaves. It is shown that both theobromine and caffeine are biosynthesized from xanthosine *via* a pathway with three steps of S-adenosyl-L-methionine (SAM)-dependent methylation in tea and coffee plants, in which methyl donors SAM and tea caffeine synthase (TCS) are the key rate-limiting factors (Kato et al., 1999). TCS, a dual-function SAM-dependent *N*-methyltransferase, catalyses the methylation of 7-methylxanthine into theobromine and methylation of theobromine into caffeine (Kato et al., 2000). RNA interference (RNAi) of *TCS* resulted in decreased caffeine and theobromine in tea leaf (Mohanpuria et al., 2011). The species in *Thea* section, with higher expression level of *TCS*, contain higher concentration of caffeine than the species in the non-*Thea* section (Xia et al., 2017).

## Conclusion

Deficiency in grana stacking in chloroplasts and low transcription of the *DXS* (*DXS1* and *DXS2)* and *POR* (*POR1* and *POR2*) gene families in the MEP and chlorophyll biosynthetic pathways lead to a reduced biosynthesis of chlorophyll and carotenoid, which results in albino phenotype of cultivar ‘Xiaoxueya’. Suppressed expressions of the *TCS* gene in the caffeine metabolic pathway and genes *CHS1*, *CHS2*, *CHS3*, *CHI*, *F3’5’H1*, *F3’5’H2*, *ANR1*, and *ANR2* in the flavonoid pathway are important factors leading to the reduced contents of alkaloids and catechins in albino leaf of ‘Xiaoxueya’, respectively.

## Author Contributions

Y-RL, Y-JY and N-NL conceived and designed the research. N-NL, Q-SL, LW, Y-CW and C-QD performed the experiments. N-NL, J-LL, and X-QZ analysed the data. N-NL and X-CW wrote the manuscript. All authors reviewed the manuscript.

## Funding

This work was supported by the National Natural Science Foundation of China (31700615), the China Postdoctoral Science Foundation (2016M600150 and 2017T100119), and the Earmarked Fund for China Agriculture Research System (CARS-19).

## Conflict of Interest

The authors declare that the research was conducted in the absence of any commercial or financial relationships that could be construed as a potential conflict of interest.
