# Supplementary material for: Dissection of Chemical Composition and Associated Gene Expression in the Pigment-Deficient Tea Cultivar ‘Xiaoxueya’ Reveals an Albino Phenotype and Metabolite Formation
Source: Front Plant Sci. 2019 Nov 27;10:1543. doi: 10.3389/fpls.2019.01543 (PMC6890721; doi:10.3389/fpls.2019.01543)
Supplement: Supplementary file 1 [file DataSheet_1.doc]

**TABLE S1**. Sequences of primers used for quantitative real-time PCR.

| **Gene name** | **Accession number of nucleotide sequence in NCBI** | **Forward primer (5’→3’)** | **Reverse primer (5’→3’)** | **Amplicon length (bp)** | **Whether the intron-spanning primers** |
| --- | --- | --- | --- | --- | --- |
| *β*-*Actin* | HQ420251 | CCTCCTCATGCTATCCTCCGTCTT | ATTTCCCGTTCAGCAGTGGTG | 113 | No |
| *DXS1* | MH925307 | CTGTGTGGAGGCAGCAAAAC | ACATGAGAGCTGAAGCCACC | 168 | No |
| *DXS2* | MH925308 | CCTAGCTGCTTCAGGTTCCC | CCTGCTGCTTCCACACATTG | 167 | Yes |
| *HDR* | JQ014628 | GCTTTTGCTGGGGTGTTGAG | CCAAAAGCAGGCAGAACCAC | 217 | Yes |
| *GGPS* | KU892076 | TCAGTGCCCAACTACACGAC | GCGAAACTGCCGCTTCTATG | 194 | No |
| *PSY* | KM519981 | GCTCGATGCTGCTTTGTCAG | AGATTCAGGCGCAATTCCCA | 199 | Yes |
| *PDS* | KF646537 | AAGGACGTGTGCCCTTTGAA | CTGCACCAGCAATGACAACC | 154 | Yes |
| *ZDS* | KM519982 | GATACAACGGCTGGGTCACA | AGCAATGAGCCTTGTCCCTC | 172 | Yes |
| *LCYE* | HM536196 | CGATCACTGTCAGAGGCTCC | CTTGCGGCCAAAGAGTGTTC | 139 | Yes |
| *LCYB* | KM519983 | GACACTACCTGGTCTGGTGC | AAGAACTACGGCGGCTTGAA | 231 | No |
| *LUT1* | KM519984 | GGCTTGTTGCTGAGGTCTCT | AGCAGAGCCATTGAGTGCAT | 200 | Yes |
| *ZEP* | KM519985 | TGCTCTTGAGCGTGCTGTTA | GTTCGCTCCGCAAATCTGTC | 239 | Yes |
| *VDE* | KM519986 | CGAATACGGACTCCAGACGG | GCGTCATTCCTGCCTCGATA | 200 | Yes |
| *NCED1* | KM519987 | ATCCGTCATCGTTGTCTCCG | CAATGGGTTTGCTCCGTTCC | 251 | No |
| *NCED2* | KM519988 | GAGAAGGCGTGGAAATCGGA | CTTCGCTGTAATGACGGCAC | 254 | Yes |
| *EARS1* | MH925311 | CAAGGCATTGTGCGAAGAGG | AAAAGGTTTCTCCGGCCCAT | 210 | Yes |
| *EARS2* | MH925312 | AAACGGCATGGTGCAACTTC | CAGTGCTCTTCCAGTGCTCA | 277 | Yes |
| *HemB* | MH981240 | CCCTGCGCTTCCACTTCATA | GGCCTTTGACACCTCTTCCA | 208 | Yes |
| *HemE1* | MH981241 | ACATTCGCTTCTGTTCCCCA | CCAGCCCTCGAACAGCATTA | 127 | Yes |
| *HemE2* | MH981242 | CAACTTCCCTCGGGCTTCTT | TCCCAGGGCTGCAAAGAAAT | 216 | Yes |
| *HemH* | MH981238 | AGAGTTGGGCCAGTGGAATG | TTCACAACCCAGTGCAGGAA | 210 | No |
| *CHLH* | HQ660368 | AGTTTTGGCTTGGTGGGTCA | ATACATGGGGCCAAAGGGTG | 154 | No |
| *CHLD* | KM519989 | TGCCACTGCAACTCTCGAAT | CTGTTTTGGCTGTTCCTCGC | 203 | Yes |
| *CHLI* | MH981239 | GGTGTCAAGGCATTTGAGCC | CACTGTCCCGACTTGAGCAT | 264 | No |
| *POR1* | MH925309 | GCTGAAACAGGCAAATGGCA | GACAGCAGCATTGCAAACCA | 204 | No |
| *POR2* | MH925310 | GCAATGCTGCGGTCTACTTG | AGGTTAGCCTTTGGAGGCAC | 223 | Yes |
| *CAO* | HQ660369 | CCAAGGGATGGAGTGTTCCC | ATGGGAGCAAAATCCAGCGA | 265 | Yes |
| *CHLP* | KM519990 | AGTACATCGGAATGGTCCGC | GATCGACTTAGCCACTCGGG | 248 | No |
| *CHLG* | KM519991 | TCCATTGGTTTGGGGAGTGG | CCTCCTAAAAGCAGCACCCA | 249 | Yes |
| *SAMS1* | MH981235 | GGCCCGATGGAAAGACTCAA | TGACAAATCGACCTGACGGG | 225 | No |
| *SAMS2* | MH981236 | GAGATCACCACCAAGGCCAA | TCAGGGGTTTCATCAGTGGC | 242 | No |
| *SAMS3* | MH981237 | GCTGGTCTCACTGGTCGAAA | ATGGCATACGAGACCTGCAC | 191 | No |
| *SAMS4* | KF750636 | GGAGTCTGTGAACGAGGGAC | ACCTTGGGCAATATCAGGGC | 295 | No |
| *XDH* | JN650203 | ATCGATGGAAGAAGCGTGGG | GAAGCAGCAACCTGAGCAAC | 184 | Yes |
| *TCS* | AB031280 | AGTGGACTTGGGTTGTGCAG | AAAGACCCCGGTACTCCCAT | 234 | No |
| *UAZ* | MH981234 | TCGCCACCGATACCATGAAG | AATGCCAGAAGTCACCAGCA | 275 | Yes |
| *GS1* | AB117934 | CCTCAGAAGCAAAGCCAGGA | TGTTGGGATTGGCTCTCCAC | 217 | Yes |
| *GS2* | AB115183 | ATGAATTGTGGGTGGCTCGT | TGCCTTCACCATAAGCAGCA | 228 | Yes |
| *GS3* | MH981231 | AAACGCTACAAGGCTGCTGA | AATGACTTATCCGCTCCGGC | 185 | Yes |
| *GOGAT1* | MH981232 | TCGGAAAAACTGGGCTTGGA | ATCTTGTTGCCTCTTGCCCC | 237 | No |
| *GOGAT2* | MH981233 | TTTGAGCTACCACAGCCAGG | AACGCCAGCCAAGAACAGTA | 136 | Yes |
| *GAD1* | KX485376 | TTCGCTTCTCGATACGTCCG | AGCTTATCGCACTCTGGCTC | 173 | No |
| *GAD2* | KX485377 | CATGGGCATGGAGGGTTACA | GTCATGTGCTCGACATTGGC | 246 | No |
| *GAD3* | KX485378 | GCTTGGTGTTCGACATCTGC | CAGTGATCTCCCTCTGCGTC | 138 | No |
| *TS* | JN226569 | CCAGGACCTCAGGGACCATA | GATGTAACGAGCCACCCACA | 210 | Yes |
| *AlaAT* | KU925842 | TGATGAAGCAACAGGGTGGG | GTCCTGTTGGATTGCCTGGA | 122 | No |
| *PAL1* | KY615669 | GAACCCCTTGATGCCACTGA | CAATTTGGCCAGGATGGTGC | 259 | No |
| *PAL2* | KY615671 | CCAATCATGTCCAAAGCGCC | ACACCCATCGTTAGAGTGCG | 226 | No |
| *PAL3* | KY615672 | CAATTCCAAGGCAGTTGGGC | TGGCAAGGATGTTGGCTTCA | 182 | No |
| *PAL4* | KY615670 | CAAAGCACCGAGCAACACAA | TCCACCACTGTGAGCAAGTC | 272 | No |
| *4CL1* | KY615680 | TCGATTCGGACGGTGATGTC | GCACCAGTGTCAGGGTCTAC | 233 | Yes |
| *4CL2* | KY615678 | TCAGCAGGAGAGGTTCCAGT | GCTCGCAAGTCCTTCCTCAA | 182 | Yes |
| *4CL3* | MH981229 | CAGGGCTTTGGTATGACGGA | TGAGTTTGGTGGCTTGTGGA | 226 | Yes |
| *CHS1* | KC357707 | TAGGCAGGACATGGTTGTGG | TGAGTCGCTTGACTGATGGG | 194 | No |
| *CHS2* | KC357708 | GGCCGATTACCAACTCACCA | TGGCCCACAAGGCTATCAAG | 219 | No |
| *CHS3* | KC357709 | CGCGTGCGTGCTATTCATAC | CCCAAATAGCACACCCCACT | 100 | No |
| *CHI* | KY615684 | CCTCCGACTGTGAAACCTCC | ACACTCCGATAGCCGTGAAC | 112 | Yes |
| *F3H1* | KY615689 | CGGTGCAAGACTGGAGAGAG | CCTTCGTCAGTGCCTCCTTC | 198 | Yes |
| *F3H2* | MH981230 | TCCTGAATGGCCCTCAAACC | CTGCCATGTGTTGTCCTTGC | 167 | Yes |
| *F3’H* | KY615695 | GATGCTGATGGTGAGGGAGG | TTCGAGGGAGAGAGAGTGGG | 286 | Yes |
| *F3’5’H1* | KY615696 | AGCGTGGGATGAAGAGGTTG | GCTTGATGATGTGTCGGTGC | 218 | Yes |
| *F3’5’H2* | KY615697 | AGACCCTGATGTGTGGGAGA | ATCATTGCTGAAAGCGGCAC | 282 | No |
| *DFR* | KY615690 | AGAAAGCAGCATGGGAAGCA | GCCTGAGGACGCTCATACAA | 229 | Yes |
| *LAR1* | KY615698 | AGCAGCAGCAGCAGTGAATA | TCTGGGCAGTGTTTCCATCC | 233 | Yes |
| *LAR2* | KY615699 | GGCCCTACCATGACAACACA | AGCAGCGGCAAGTAAGTCAT | 284 | Yes |
| *ANS1* | KY615704 | AAGCGAGTACGCAAAGCAAC | GGATGAAGGTGAGGGCAGAG | 218 | No |
| *ANS2* | KY615703 | ACCGACTACATTCCTGCGAC | CATGCTGGGGAGGATGAAGG | 249 | Yes |
| *ANR1* | KY615701 | ATAAGCTCAATGGGACCGGC | GGCCATGAGAGTTGGGATGA | 185 | Yes |
| *ANR2* | KY615702 | TGCCACCCTTGTGAAGATGT | TCCAGTGCTACGAGGTGAGA | 102 | Yes |

**TABLE S2.** Sequences of primers used for subcellular localization.

| **Gene name** | **Accession number of nucleotide sequence in NCBI** | **Forward primer (5’→3’)** | **Reverse primer (5’→3’)** |
| --- | --- | --- | --- |
| *DXS1* | MH925307 | CGGGATCCATGGGTTCTTGTGGTGTTATCAAGAGC | ACGCGTCGACGAGGTTGAGAAGGTGAAGGTTTTC |
| *DXS2* | MH925308 | CGGGATCCATGGCTGTCTCGGGGCTTGTC | ACGCGTCGACCTTGAACTTGAGAGCTTCTTTGGATTT |
| *POR1* | MH925309 | CGGGATCCATGGCTCTCCAGGCTGCTGCT | ACGCGTCGACTGCCAGACCCACAAGTTTCTC |
| *POR2* | MH925310 | CGGGATCCATGGCTCTCCAAGCAACTTCTTTA | ACGCGTCGACAGCCAATCCGACAAGCTTCTC |

**
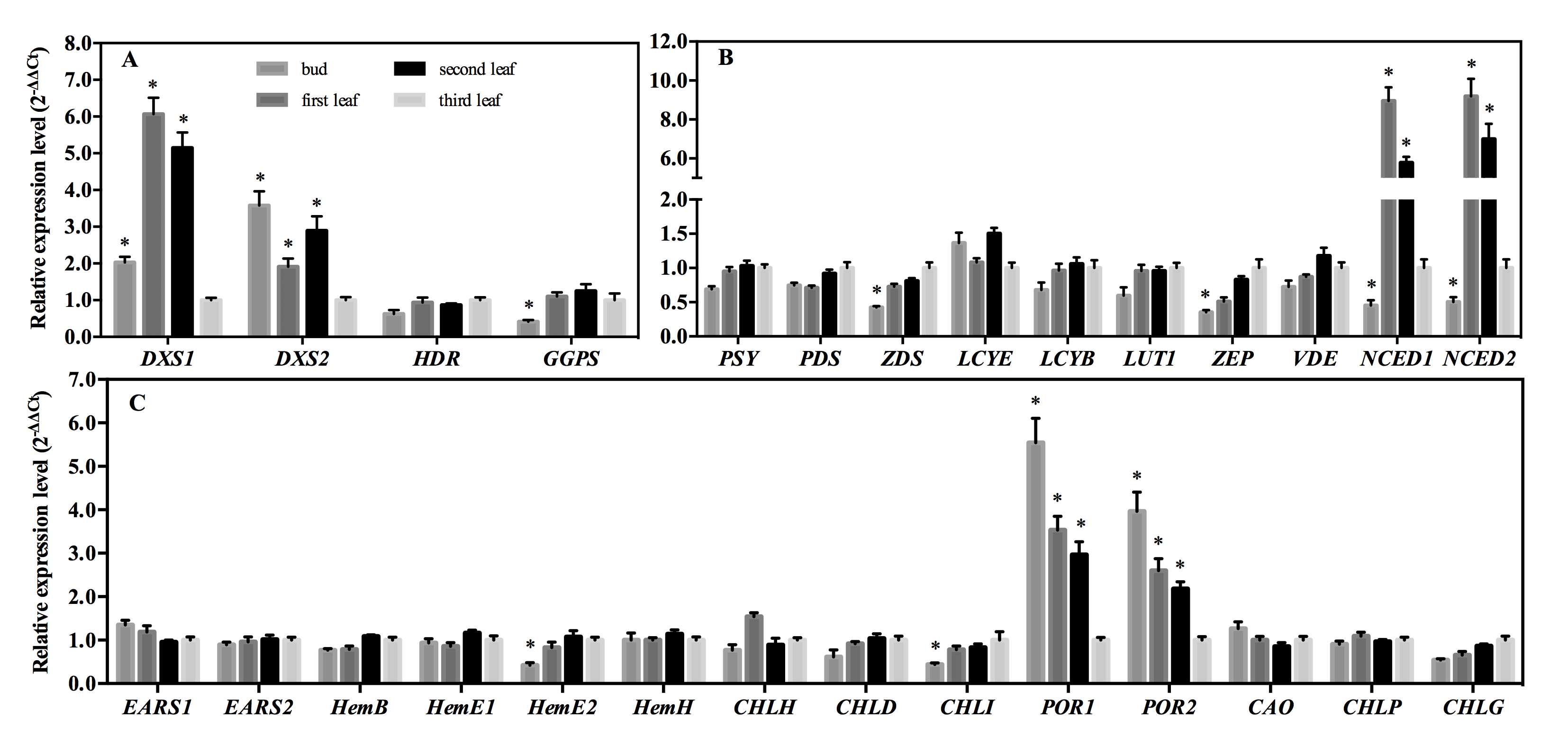
**

**Figure S1.** Relative expression levels of genes related to pigment metabolism in ‘Xiaoxueya’ leaves. (A) Genes involved in the MEP biosynthetic pathway. (B) Genes involved in the carotenoid biosynthetic pathway. (C) Genes involved in the chlorophyll biosynthetic pathway. Data are presented as the mean of three replicates (± standard deviation). Asterisks mark columns with significant differences (*p* value ≤ 0.05) and |log2FoldChange| ≥ 1 between the values from the sample and third leaf.


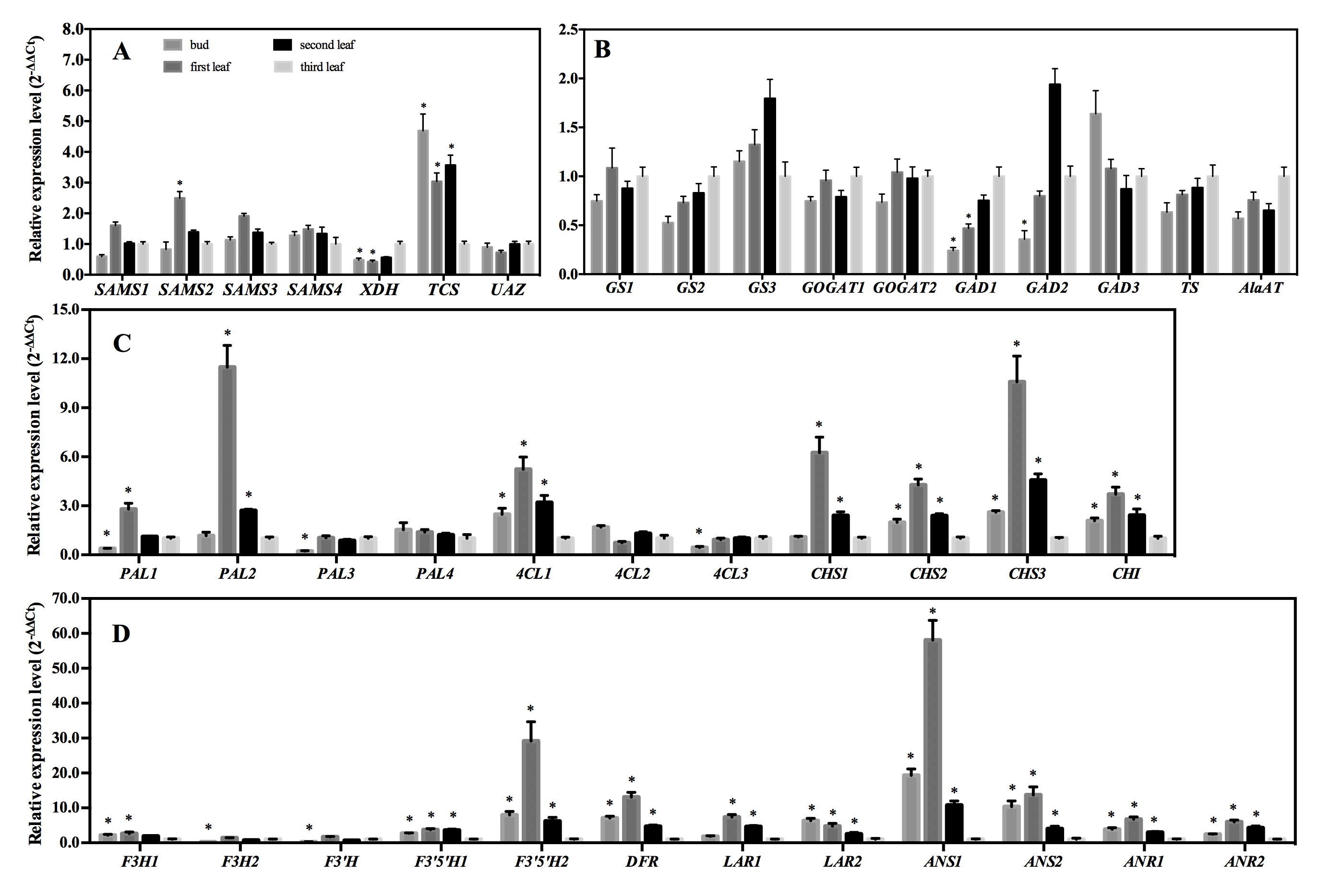


**Figure S2.** Relative expression levels of genes related to quality metabolites in ‘Xiaoxueya’ leaves. (A) Genes involved in the caffeine biosynthetic pathway. (B) Genes involved in the theanine biosynthetic pathway. (C, D) Genes involved in the catechin biosynthetic pathway. Data are presented as the mean of three replicates (± standard deviation). Asterisks mark columns with significant differences (*p* value ≤ 0.05) and |log2FoldChange| ≥ 1 between values from the sample and third leaf.
